# Integration of sociocultural and behavioral factors into the clinical framework of cardiovascular studies in Hispanic/Latino populations: Relevance during the SARS-COV-2 pandemic

**DOI:** 10.1017/cts.2021.20

**Published:** 2021-03-08

**Authors:** Shakira F. Suglia, Ana F. Abraido-Lanza, Rafael E. Guerrero-Preston, Kenneth S. Ramos

**Affiliations:** 1 Department of Epidemiology, Rollins School of Public Health, Emory University, Atlanta, Georgia; 2 School of Global Public Health, New York University, New York, USA; 3 Department of Biomarkers Discovery and Development, LifeGene Biomarks, San Juan, Puerto Rico; 4 Texas A&M Institute of Biosciences and Technology, Houston, USA

**Keywords:** Socio-cultural, behavioral, clinical studies, Hispanic/Latino, SARS-COV-1

## Abstract

Recent reports on the burden of cardiovascular disease (CVD) in the USA indicate that despite significant declines in CVD mortality in the late 20th century, this decline is now decelerating and may be worsened by inequalities in health care. Social factors contribute to most of the cardiovascular health disparities documented to date. Hispanics/Latinos and African-Americans share a higher prevalence of cardiovascular risk factors and experience higher rates of poverty and social stressors than non-Hispanic Whites. We propose that the use of social and behavioral data beyond basic and sometimes loose identifiers of race/ethnicity, educational attainment, and occupation would inform clinical practice and greatly facilitate the provision of adequate guidance and support to patients regarding continuity of care, adherence to medications and treatment plans, and engagement of participants in future research. This perspective briefly highlights factors deemed to be critical for the advancement of Hispanic/Latino health and delineates pathways toward future applications.

## Introduction

Recent reports on the burden of cardiovascular disease (CVD) in the USA indicate that despite significant declines in CVD mortality in the late 20th century, this decline is now decelerating [1], and may in fact be showing early signs of reversal [2]. In this context, it has been suggested that the prevalence of CVD in the USA may increase between 2010 and 2030 due to inequalities across different racial and ethnic groups [3]. The reasons for increased CVD prevalence and deceleration in CVD mortality are multifactorial. Of note is the increased prevalence of obesity and its association with increased prevalence of hypertension, diabetes, and other CVD risk factors. Along with the rising obesity epidemic, inequalities in healthcare delivery, access to care, and other social and environmental disparities have continued to widen across the spectrum of income groups [4]. Social factors, broadly defined as “the circumstances in which people are born, grow, live, work, and age and the systems put in place to deal with illness, contribute to most of the CVD disparities documented to date”[5]. These inequities have recently come “closer to home” by the overt disparities in SARS-CoV-2 burden among Hispanics/Latinos and African-Americans compared to non-Hispanic Whites [6].

Hispanics/Latinos are the largest ethnic minority, comprising an estimated 17.8% of the population, while African-Americans make up 12.7% of the population [7]. These two minority groups have a high prevalence of cardiovascular risk factors and experience higher rates of poverty and social stressors [8]. The term Hispanic/Latino is the dominant term used to describe people in the USA that trace their ancestral roots to Spain/Latin America. More recently, the gender-neutral term “Latin(x)” has emerged as an alternative to describe the Hispanic/Latino population, though its use is not yet widespread. The American Heart Association statement on social determinants of risk and outcomes for CVD postulates that “the most significant opportunities for reducing death and disability from CVD in the US lie in addressing the social determinants of cardiovascular outcomes” [9]. Incidentally, Hispanics/Latinos have lower CVD mortality than their African-American and White counterparts [10], and this paradoxical finding cannot be explained by behavioral factors alone. Thus, there is a pressing need to better understand how social and behavioral determinants of health contribute to CVD outcomes across different demographic groups as well as within the various Hispanic/Latino subgroups.

### Social and Behavioral Determinants of Health

Social determinants of health include economic, environmental, interpersonal, and health-related constructs that directly influence health outcomes. In addition, psychosocial and sociocultural factors are key determinants of health across the life course. In spite of their importance, these social and behavioral factors are not routinely documented during a clinical encounter. While the National Academy of Medicine has issued recommendations for the collection of social determinant measures in electronic health records [11], the collection of these data varies extensively across different areas of medical practice. Also noteworthy is that even in cases when the data is collected at point of care, the findings are either not properly captured or poorly documented in the electronic health record. Consequently, the clinical utility of social and behavioral data is grossly underutilized in current clinical practice.

The use of social and behavioral data beyond basic and sometimes loose identifiers of race/ethnicity, educational attainment, and occupation would not only inform clinical practice, but facilitate the provision of adequate guidance and support to patients regarding continuity of care, adherence to medications and treatment plans, and engagement of participants in future research. In this regard, we posit that there is a critical need to elucidate pathways through which social and behavioral factors “get under the skin” to modify biology in ways that directly impact health outcomes. Arguably, the integration of social and behavioral data into routine clinical practice would provide a more holistic view of the patient, as well as a clearer understanding of the critical elements that impact physical health. This perspective briefly highlights factors deemed to be critical for the advancement of Hispanic/Latino health and delineates pathways toward future applications.

### Need for Focused Studies of Hispanics/Latinos

Originating from over 20 countries in Latin America and the Caribbean, Hispanic/Latino subgroups in the USA have diverse sociodemographic, genetic, and health profiles. As of 2018, the US Census Bureau estimated that nearly 60 million Hispanics/Latinos currently live in the USA, comprising 18% of the overall population [12]. Although “Hispanics/Latinos” constitute diverse, heterogeneous populations, much research focuses on Hispanics/Latinos as a pan-ethnic, homogenous group. The major Latino groups in the continental USA include Mexicans (63.0%), (mainland) Puerto Ricans (9.2%), and Cubans (3.5%). The fourth largest group, Salvadorans, represents 3.3% of all Latinos, but this group is typically combined with others in the “Central and South American” category. Individuals from the Dominican Republic, typically fall into the “Other Hispanic” category, and constitute the 5th largest subgroup in the nation, representing 2.8% of all Hispanics/Latinos [13], In addition, there are roughly 3.5 M US citizens of Puerto Rican origin residing in the island of Puerto Rico [14]. There are clearly significant differences between the various Hispanic/Latino groups including country of origin, dominant cultural influences, and varying degrees of genetic ancestry. For example, the various Latino groups differ in nativity status [15], where roughly one-third (36%) of Mexicans in the USA are foreign-born. In contrast, more than half of Cubans (59%) and Dominicans (57%) and approximately two-thirds of Salvadorans (62%) are foreign-born. Among Puerto Ricans living in the continental USA, one-third (31%) were born on the island [16].

The HCHS/SOL, the largest study of Hispanics/Latinos in the USA, noted stark differences in cardiometabolic health profiles among Hispanics/Latinos of differing backgrounds. In one study by Davigllus et al. [17], over 29% of Puerto Rican women were diabetic compared to 19% of Mexican women. Other conditions such as asthma, also vary greatly among Hispanics/Latinos, with Puerto Rican children having the highest rates of asthma compared to any other racial/ethnic group in the USA and Mexican American children having asthma rates comparable to non-Hispanic whites [18]. In this case, evidence has implicated psychosocial stress in asthma morbidity among Puerto Rican children, a relationship that may be linked to variations in specific genes1 [19]. Although there is growing recognition of differing health patterns and profiles among the various Hispanic/Latino groups, there are almost no data on health or health behaviors of Salvadorans, very few studies of Dominicans, and practically no understanding of the biological and social underpinnings responsible for any differences between these and other Hispanic/Latino groups. While extensive work has documented a relationship between sociocultural and behavioral factors as they impact health outcomes, little research has focused on the mechanisms by which these factors influence health. For example, acculturation, defined as the process through which an immigrant population assimilates to a new dominant culture, has been associated with both better and worse health outcomes [20]. To date, there is almost no research testing the mechanisms by which acculturation may affect general health and various health behaviors [21]. Greater acculturation may lead to changes in health-related and other beliefs, factors unrelated to health beliefs, or to changes in norms or values that may directly or indirectly impact health behaviors. Another hypothesized mechanism involves the regulation of stress pathways which in turn connect to biological mechanisms. There is an expansive literature on the effects of stress on mental and physical health outcomes among racial–ethnic minorities including Hispanics/Latinos, and mounting evidence that minorities are more likely to be exposed to stressors than nonminority populations [22]. Chronic stress associated with low socioeconomic status, toxic neighborhoods, adverse childhood events, psychosocial traits, and lifestyle choices can influence health trajectories and disease risk in Hispanic/Latino populations. These stressors differentially modulate biological pathways that intersect with environmental and lifestyle factors to define health outcomes. Thus, a clear understanding of modifiable and non-modifiable factors needs to be developed.

### Getting Under the Skin

Health and disease can be modulated via biological mechanisms linked to molecular alterations in the epigenome, the set of chemical modifications to DNA (e.g., methylation and hydroxymethylation), and/or to the histone proteins that closely associate with DNA (e.g., methylation, acetylation, phosphorylation). Epigenetic modifications are critical determinants of when genes are switched on or off as well as the timing of gene expression. This is best exemplified by the growing body of evidence implicating socioeconomic stressors and low childhood socioeconomic status as regulators of DNA methylation and altered expression of stress-related and inflammation genes [23]. Thus, stress-regulated epigenetic changes may be key mechanisms by which the social environment, lifestyle, and behaviors “get under the skin” to become biology. Similar relationships may govern interactions between the microbiome and stress-regulated biology. More research is needed at the intersection of biology and psychosocial domains to define how social and economic stressors reversibly and non-reversibly alter biology and the degree to which these interactions depend upon life stages and other factors.

Big data analytic strategies using multilevel approaches are already in place using machine learning and computer algorithms to integrate patient demographic, psychosocial, clinical, pathology, and molecular profiles at the individual level, treatment recommendations, health insurance coverage, clinical, and health information data at the hospital or medical center level, and publicly available geocoded socioeconomic, environmental, social determinants of health data at neighborhood levels [24]. The multilevel integration of multiple data streams can track the health information and status of individuals from cradle to grave, using mathematical modeling similar to the ones used by air traffic controllers to track air traffic and follow the path of a flight. These elements can help define a framework to effectively elucidate how social, biological, and psychological processes determine epigenomic changes and to combine multiple data streams into next-generation precision medicine tools. These approaches will allow precise depictions of molecular, clinical, psychosocial, and contextual portraits for tracking health trajectories across the lifespan.

A recent National Academies report provided specific recommendations on the candidate set of social domains that may be considered for incorporation into the electronic health record [25]. The critical domains included sociodemographic, psychological, behavioral, individual, and neighborhoods and communities. A review of the specific elements captured within each of these domains indicates that they are informative and necessary to improve the quality of holistic health care provided. For Hispanic/Latino populations, we posit that all these domains are critical given the large degree of heterogeneity that characterizes the different subgroups, and the general lack of understanding of how known differences among the various groups translate into health and health outcomes.

### Moving Forward

As platforms are put in place to guide future efforts in the study of Hispanic/Latino health, efforts should focus on improved stratification of Hispanic/Latino populations that facilitate the identification of elements shared by the different subgroups as well as the key differences that must be incorporated into risk assessments, risk communication, prevention modalities, and clinical management. These efforts can help the medical and public health communities to not only address the growing divide between majority and minority health issues, but also to increase the precision with which modalities are applied to deal with the heterogeneity of Hispanic/Latino populations. This challenge is best exemplified by recent reports showing that Hispanics/Latinos and African-Americans are being disproportionately impacted by the spread of the SARS-COV-2 virus [26]. This viral infection has become a major public health emergency due to its high morbidity and mortality. Infection has been associated with multiple organ dysfunctions, including major cardiovascular abnormalities [27].

Social and behavioral influences on health status, comorbidities, working and living conditions, and poverty lie at the core of the disproportionate burden of illness and death. It remains to be determined, however, the degree of heterogeneity among the Hispanic/Latino population both in terms of incidence and clinical outcomes. For instance, it is not yet known if there are differences in COVID-19 mortality between native-born Hispanics/Latinos and immigrant populations, or whether isolation from government and healthcare systems due to language barriers, socioeconomic status, or documentation status are the primary determinants of worsened health outcomes [28]. Systematic assessment is needed on the degree to which living conditions, population density, and housing segregation may impact the ability of minority populations to practice social distancing. Further, the types of occupation and demands placed on minority workers may also pose heightened risks of exposure to the virus [29]. For example, some analyses indicate that Hispanics/Latinos and African-Americans are overrepresented in “front-line” occupations in New York City, which was cited as the epicenter of coronavirus in the USA during the early months of the pandemic [30]. These socioeconomic determinants likely will continue to magnify and widen disparities in health-related outcomes to coronavirus.

As a way of framing and understanding the impact of biological, social, and behavioral determinants of health, an ecological approach that considers the individual within a sociocultural context may be particularly useful. The ecological approach considers a number of interacting and interdependent contextual influences on health including individual and interpersonal contexts (e.g., coping strategies, relationships between two or more individuals), social and cultural contexts (e.g., socioeconomic status, level of acculturation, or cultural values), and structural contexts (e.g., neighborhood quality, community cohesion, federal or state policies and laws). In addition, the temporal contexts (e.g., life or developmental stage) should also be considered.

A case is made here for the need to gain a deeper understanding of the heterogeneity of Hispanic/Latino populations, differences that go far beyond the country of origin and that encompass a diversity of factors best captured by the inclusion of multiple biological, social, and behavioral determinants of health. This approach would allow us to go beyond documentation of what groups are at highest/lowest risk of certain conditions, to a better understanding of why risk differs across subgroups and how best to devise appropriate interventions.
